# Neonatal sepsis: any role for procalcitonin as a diagnostic marker?

**DOI:** 10.1186/s12887-026-06576-1

**Published:** 2026-03-30

**Authors:** Ogechi Ezerioha, Joseph Ezeogu, Emeka Nwolisa

**Affiliations:** Department of Paediatrics, Federal Teaching Hospital, Owerri, Imo State Nigeria

**Keywords:** Neonatal sepsis, Procalcitonin, Biomarker, Diagnostic accuracy, Early-onset neonatal sepsis, Late-onset neonatal sepsis

## Abstract

**Introduction:**

In low and middle-income countries, a key driver of neonatal morbidity and mortality is neonatal sepsis. Rapid and precise diagnosis of neonatal sepsis remains a critical challenge due to limited laboratory facilities. Blood culture results, the diagnostic gold standard, are often unavailable or delayed; therefore, procalcitonin (PCT) has been considered as a potential inflammatory biomarker of bacterial infection.This study assessed the diagnostic performance of PCT in neonates with sepsis at the Federal Teaching Hospital, Owerri, Nigeria.

**Methods:**

This cross-sectional study recruited 75 neonates aged 0–28 days with clinical suspicion of sepsis between June 2023 and February 2024. Sociodemographic and clinical data were obtained using a structured questionnaire, serum PCT levels were measured using Enzyme-linked immunosorbent assay (ELISA) and blood culture was performed as the gold standard. The primary outcome was the diagnostic accuracy of PCT for neonatal sepsis. Secondary outcomes included comparison of diagnostic performance between early-onset neonatal sepsis (EONNS) and late-onset neonatal sepsis (LONNS), and description of microbial isolates. Diagnostic indices (sensitivity, specificity, positive predictive value (PPV), negative predictive value, NPV) were calculated at different PCT thresholds. Receiver operating characteristic (ROC) curves were analysed to determine the area under the curve (AUC).

**Results:**

Of the 75 neonates, 55 (73.3%) had culture-confirmed sepsis and 32 (58.2%) early-onset neonatal sepsis. Serum PCT levels were not normally distributed (Kolmogorov–Smirnov test, *p* < 0.001) and were summarized using medians and interquartile ranges. At a PCT cut-off of ≥ 2 ng/mL, sensitivity and NPV were 72.7% and 57.1% respectively while specificity and PPV remained 100% for all neonates. The AUC for PCT was significantly higher in EONNS (0.964, 95% CI: 0.910–1.000) compared to LONNS (0.788, 95% CI: 0.638–0.938). Gram-negative organisms (63.6%) were the most common isolates, mainly *Escherichia coli* (34.5%).

**Conclusion:**

Serum procalcitonin demonstrates high specificity for culture-confirmed neonatal sepsis at a cut-off of ≥ 2.0 ng/mL in this cohort, with better diagnostic performance in early-onset disease. These findings apply to the studied population and cut-off thresholds. PCT measurement integration in Nigeria can be a priceless tool for guiding rational antibiotic therapy; particularly where rapid confirmation of sepsis is critical to improving neonatal survival outcomes.

## Introduction

Neonatal sepsis remains an important cause of morbidity and mortality, particularly in low- and middle-income countries [1]. Globally, a quarter of all 2.4 million neonatal mortalities were attributable to neonatal infections/sepsis, with the highest burden (27/1000 live births) in Sub-Saharan Africa and Nigeria accounting for the highest rate in Africa [2]. Neonatal sepsis is a systemic infection occurring within the first 28 days of life, often classified as early-onset (within 72 h of birth) or late-onset (after 72 h) [3]. Its clinical presentation is often non-specific, characterized by systemic signs and symptoms that overlap with non-infectious conditions during the first 28 days of life [1, 3]. This makes diagnosis difficult and delayed. Blood culture, the diagnostic gold standard, is often inaccessible or unaffordable and may yield false negative results due to prior antibiotic exposure or low bacteremic load [2], a turnaround time of 24–48 h leading to delays in treatment or unnecessary antibiotic use [4]. This highlights the critical need for reliable, rapid biomarkers to aid early diagnosis of neonatal sepsis and antibiotic stewardship.

Procalcitonin (PCT), the precursor peptide of calcitonin, has emerged as a potential specific biomarker for bacterial infections. Levels, which are very low (< 0.05 ng/mL) in healthy individuals, rise rapidly within 2–4 h of a bacterial challenge due to stimulation by endotoxins and cytokines (IL-6, TNF-α) [5, 6]; and is detectable in the serum for up to seven days. Furthermore, viral infections typically do not elicit a strong PCT response, enhancing its specificity for bacterial sepsis [6]. It has also shown promise in adult and paediatric sepsis protocols. The diagnostic performance of a biomarker is optimally assessed using receiver operating characteristic (ROC) curve analysis, which plots sensitivity against 1-specificity across various thresholds [7], and the overall accuracy is expressed as AUC. The greater the AUC, the better the test [8]. While PCT has been extensively studied in high-income settings; data from Nigerian neonatal populations are limited.

In addition to biological biomarkers such as procalcitonin, emerging evidence suggests that computational tools, including general-purpose large language models, may also assist clinicians in identifying infants at risk of serious bacterial infection, as demonstrated in a recent study [9]. It is pertinent to state that the growing interest in non-invasive diagnostic approaches also highlights the potential value of salivary biomarkers in paediatric infectious and inflammatory conditions. Recent evidence, demonstrates that salivary biomarker profiling can offer clinically meaningful diagnostic information while avoiding the burden of blood sampling, an advantage of particular relevance in vulnerable neonatal populations [10]. Although promising, salivary biomarkers for neonatal infectious diseases remain largely investigational and are not yet clinically validated for routine sepsis diagnosis.

This study therefore aimed to evaluate the diagnostic accuracy of serum PCT as a screening marker for neonatal sepsis, using blood culture as the reference standard at the Federal Teaching Hospital Owerri, Nigeria. Secondary objectives were to determine the optimal procalcitonin cut-off values, compare its diagnostic performance in early-onset and late-onset neonatal sepsis, and describe the bacteriological profile of sepsis seen in the study population.

## Method

### Study design and setting

This cross-sectional study was conducted at the Special Care Baby Unit of the Federal Teaching Hospital, Owerri, Nigeria between June 2023 and February 2024. The hospital is a 624-bed tertiary facility in the Southeastern region of Nigeria; it serves as a referral center for other adjoining states.

#### Inclusion criteria

1. Term babies (born between 37 weeks, 0 days and 41 weeks, 6 days), aged 0–28 days.
2. Neonates with clinical features suggestive of sepsis, such as.
  Temperature instability with axillary temperature > 38.5°C or < 36°C.
  Tachycardia or bradycardia.
  Refusal to feed, feeding intolerance, lethargy, excessive irritability, high pitched cry, seizures, apnoea, respiratory distress, poor perfusion, tachypnea, abdominal distension, necrotizing enterocolitis, vomiting).
  Eligible neonates presenting during the study period were consecutively enrolled irrespective of prior antibiotic exposure.

#### Exclusion criteria

Neonates with congenital anomalies, whose clinical features of sepsis have lasted more than 1 week (PCT is detectable for up to 7 days) [11] or those whose mothers declined consent.

### Sample size calculation

For diagnostic accuracy studies where sensitivity is the primary outcome, the sample size can be calculated using the formula described by Buderer (1996) [12].

$$\mathrm{Formula}:\;\mathrm{n}=\;\mathrm{Z}^{2}\times\mathrm{S}\;\times\left(1-\mathrm{S}\right)/\;\mathrm{d}^2$$

Where:

- n = minimum required sample size.
- Z = standard normal deviate corresponding to 95% confidence level = 1.96.
- S = anticipated sensitivity of the diagnostic test.
- d = desired precision (margin of error).

Values used in this study

- Anticipated sensitivity (S) = 89% = 0.89.
- from Arowosegbe et al.) [13].
- Precision (d) = 10% = 0.10
- Confidence level = 95% → Z **=** 1.96.

Substitution and calculation

$$\mathrm{n}=\left(1.96\right)^2\times{0.89}\times\left(1-0.89\right)/\left(0.10\right)^{2}=37.6$$

This is the minimum number of neonates with culture-confirmed sepsis required to estimate sensitivity with the stated precision. Given that neonatal sepsis cannot be predicted with certainty at recruitment and to ensure adequate representation of both culture-positive and culture-negative neonates, the sample size was adjusted upward. Additionally, allowance was made for possible attrition, incomplete data, and subgroup analyses (EONNS vs. LONNS). Therefore, a total of 75 neonates were recruited.

### Data and sample collection

Sociodemographic and clinical data were collected using a structured questionnaire [14]. 

#### Blood culture analysis

While observing asepsis, 2 mL of blood was drawn for culture and dispensed into a BacT/Alert^®^ PF Plus culture bottle (bioMérieux, USA). The culture vials were loaded immediately into the BACT/ALERT^®^ 3D machine according to the manufacturer’s instructions and were incubated for five days.

#### PCT analysis

A separate blood sample was collected in a plain red-top tube without anticoagulant for procalcitonin (PCT) analysis. Serum was separated by centrifugation and stored at − 80 °C until batch analysis using an enzyme-linked immunosorbent assay (ELISA; AccuBind^®^). PCT measurements were performed in batches using AccuBind^®^ PCT ELISA reagent kits (Monobind Inc., Lake Forest, CA, USA) on an AutoPlex ELISA analyser. Laboratory personnel were blinded to blood culture results. The assay was conducted strictly according to the manufacturer’s instructions. Independent determination of inter-assay and intra-assay coefficients of variation was not performed in the study laboratory; however, manufacturer-reported inter-assay and intra-assay coefficients of variation for the AccuBind^®^ PCT ELISA are < 10%, consistent with acceptable analytical performance for clinical research assays.

#### The test procedure for PCT assay

Before proceeding with the assay, all reagents, reference calibrators and controls were brought to room temperature (20–27 °C).

1. Microplates’ wells for each serum reference calibrator, control, and patient specimen to be assayed in duplicate were formatted. Any unused microwell strips were put back into the aluminium bag, sealed and stored at 2–8 °C.
2. Fifty microlitres (50 µL) of the PCT calibrator control or specimen was pipetted into the assigned well.
3. Fifty microlitres (50 µL) of the PCT Enzyme Reagent was added to all wells.
4. The microplate (Note 2) was mixed for 20–30 s until homogeneous then covered and incubated for 30 min at room temperature.
5. The contents of the microplate were discarded by decantation and the plate was blotted dry with absorbent paper.
6. Three hundred and fifty microlitres (350 µL) of wash buffer was added and decanted.
7. One hundred microlitres (100 µL) of substrate reagent was then added to all wells and incubated at room temperature for fifteen (15) minutes. All reagents were added in the same order to minimize reaction time differences between wells.
8. Fifty microlitres (50 µL) of stop solution was added to each well and gently mixed for 15–20 s.
9. The absorbance in each well was read at 450 nm (using a reference wavelength of 620–630 nm. The results were read within fifteen (15) minutes of adding the stop solution.

### Calculation of results

A dose-response curve was used to ascertain the concentration of PCT in unknown specimens.

1. The absorbance obtained from the printout of the microplate reader was recorded.
2. The absorbance for each duplicate calibrator versus the corresponding PCT concentration in ng/mL was plotted on linear graph paper.
3. The points with a best-fit curve were connected.
4. To determine the concentration of PCT for an unknown, the average absorbance of the duplicates for each unknown on the vertical axis of the graph was located, the intersecting point on the curve was found and the concentration (in ng/mL) from the horizontal axis of the graph (the duplicates of the unknown may be averaged as indicated) was read in the auto-analyser.

### Data analysis

Data were analyzed using IBM SPSS Statistics version 24.0. Categorical variables were described using frequencies and percentages, while continuous variables (like PCT) were described using medians and interquartile ranges (IQR) due to non-normal distribution. The Kolmogorov-Smirnov test was used to determine the normality or otherwise of the serum PCT levels and other quantitative data. The diagnostic performance of PCT (sensitivity, specificity, PPV, NPV) was calculated against blood culture results at different cut-off values. Receiver operating characteristic (ROC) curves were generated across multiple thresholds, and the area under the curve (AUC) was calculated to assess overall diagnostic accuracy, while diagnostic indices were emphasized at predefined cut-off values. A *p*-value < 0.05 was considered statistically significant.

### Clinical trial number

Not applicable

### Ethical approval

Ethical clearance was sought and approved on June 19th, 2022 from the Health, Research and Ethics Committee of the Federal Teaching Hospital, Owerri with number FMC/OW/HREC/VOLII/75. Parents and legal guardians of eligible neonates provided written informed consent. Data collection followed the ethical principles of declaration of Helsinki.

## Results

### Sociodemographic characteristics of study participants

Seventy-five neonates were studied, with a median age of 3 days (interquartile range [IQR]: 0.5–9 days), with a predominance of male neonates. Most of the study participants (39%) were aged ≤ 3 days at presentation. Based on the Oyedeji socioeconomic classification [15], most neonates belonged to the middle socioeconomic class. The sociodemographic characteristics are summarized in Table 1.

**Table 1:** Sociodemographic characteristics of study participants with suspected sepsis (*n* = 75)

Sociodemographic variables	*n* = 75 Frequency (%)	
Gender		
Female	24	32.0
Male	51	68.0
Median age (in days)	3	(0.5–9)
Age interval		
≤ 3 days	39	52.0
> 3 days	36	48.0
Socioeconomic class		
Upper class	13	17.3
Middle class	35	46.7
Lower class	27	36.0

### Distribution of clinical and laboratory variables of participants

The most common presenting symptoms among the study participants were fever 37 (49.3%) and difficulty in breathing 35 (46.7%). Notably, 23 neonates (30.7%) had previous use of antibiotics prior to inclusion into the study. Fifty-five (73.3%) neonates have culture-positive sepsis. Most of the study participants (53.3%) have serum PCT level ≥ 2 ng/mL. Table 2.

**Table 2:** Distribution of clinical and laboratory variables of participants

Variables		*n* = 75 Frequency (%)	
Presenting complaints			
Fever		37	49.3
Difficulty in breathing		35	46.7
Vomiting		10	13.3
Convulsion		10	13.3
Fast breathing		9	12.0
Cough		7	9.3
Abdominal distension		5	6.7
Apnea		3	4.0
Reduced spontaneous motor activity		2	2.7
Loss of consciousness		2	2.6
Inconsolable cry		1	1.3
Previous use of antibiotics		23	30.7
Blood culture positive		55	73.3
	EONNS	32	58.2
	LONNS	23	41.8
Serum Procalcitonin (ng/mL), median (IQR)		48 (24–72)	
< 0.5ng/mL		23	30.7
0.5 – < 2ng/mL		12	16.0
≥ 2 ng/mL		40	53.3

### Sepsis and procalcitonin profile

**Fig. 1:**
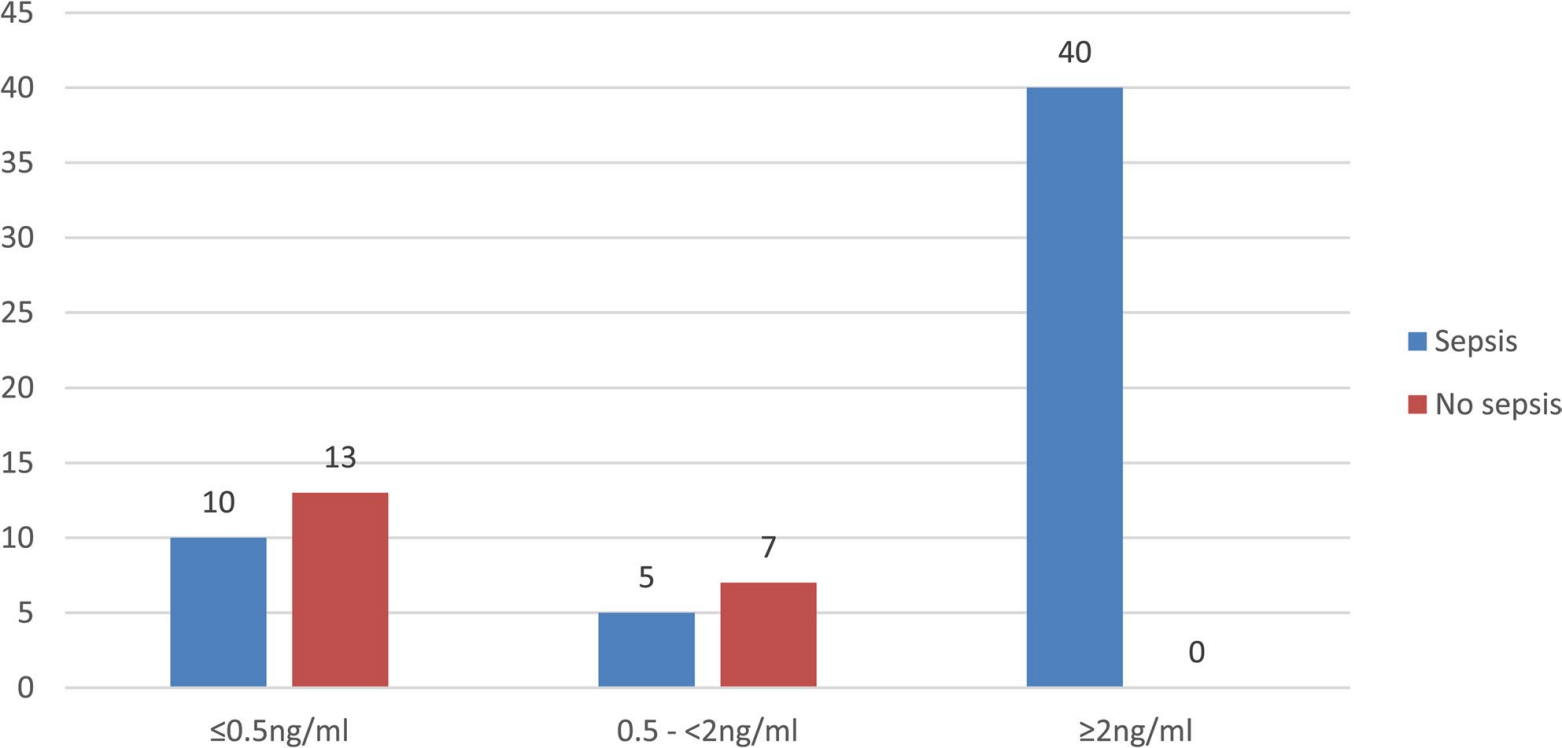
Sepsis and Procalcitonin profile

Blood culture was positive in 55 neonates (73.3%), of which 32 (58.2%) were EONNS and 23 (41.8%) were LONNS cases. The median serum PCT level was 48 ng/mL (IQR: 24–72). Majority of neonates (53.3%) had PCT levels ≥ 2 ng/mL. All neonates (100%) with PCT ≥ 2 ng/mL had culture-confirmed sepsis (Fig. 1).

### Diagnostic performance of procalcitonin at different cut-off values

Table 3 summarizes the diagnostic performance of the biomarker at different cut-off concentrations (≥ 0.5 ng/mL, ≥ 2.0 ng/mL, and ≥ 10.0 ng/mL) in neonates with suspected sepsis, stratified into early-onset neonatal sepsis (EONNS) and late-onset neonatal sepsis (LONNS).

**Table 3:** Diagnostic performance of procalcitonin at different cut-off values in neonatal sepsis

Cut-off (ng/mL)	Category	TP	FP	TN	FN	Sensitivity (%)	Specificity (%)	PPV (%)	NPV (%)
≥ 0.5	All neonates (*n* = 75)	45	7	13	10	81.8	65.0	86.5	56.5
	EONNS (*n* = 39)	30	1	6	2	93.8	85.7	96.8	75.0
	LONNS (*n* = 36)	15	6	7	8	65.2	53.8	71.4	46.7
≥ 2.0	All neonates (*n* = 75)	40	0	20	15	72.7	100	100	57.1
	EONNS (*n* = 39)	27	0	7	5	84.8	100	100	58.3
	LONNS (*n* = 36)	13	0	13	10	56.5	100	100	56.5
≥ 10.0	All neonates (*n* = 75)	18	0	20	37	32.7	100	100	35.1
	EONNS (*n* = 39)	11	0	7	21	34.4	100	100	25.0
	LONNS (*n* = 36)	7	0	13	16	30.4	100	100	44.8

Sensitivity = TP**/** (TP + FN), Specificity = TN/(TN + FP), PPV = TP/(TP + FP), NPV = TN/(TN + FN)

At the lowest threshold (≥ 0.5 ng/mL), sensitivity was highest across all groups, particularly in EONNS (93.8%), indicating strong ability to detect true cases. However, specificity was modest (65.0% overall), reflecting a higher rate of false positives. This cut-off therefore maximizes sensitivity but at the expense of specificity, which may lead to over-diagnosis.

At the intermediate threshold (≥ 2.0 ng/mL), specificity reached 100% in all groups, eliminating false positives. Positive predictive value (PPV) was also 100%, meaning all positive results correctly identified sepsis. Sensitivity, however, declined (72.7% overall), with a more pronounced reduction in LONNS (56.5%). This suggests that while the ≥ 2.0 ng/mL cut-off is highly reliable for confirming sepsis, it risks missing a substantial proportion of true cases, particularly in late-onset presentations.

At the highest threshold (≥ 10.0 ng/mL), specificity and PPV remained perfect (100%), but sensitivity dropped markedly (32.7% overall), with similar reductions in both EONNS and LONNS.

### Receiver operating characteristic curve analysis

Overall, the Area under the curve (AUC) for Procalcitonin in diagnosing neonatal sepsis was 0.899 (95% CI: 0.831–0.967, *p* < 0.001), demonstrating outstanding diagnostic accuracy. However, the diagnostic performance was better in EONNS (AUC = 0.964, 95% CI: 0.910–1.000, *p* < 0.001) compared to LONNS (AUC = 0.788, 95% CI: 0.638–0.938, *p* = 0.005) (Table 4).

**Table 4:** Area under the receiver operating characteristic curve of procalcitonin

	AUC	Standard error	95% Confidence Interval	*p*-value
EONNS	0.964	0.028	0.910–1.000	< 0.001
LONNS	0.788	0.077	0.638–0.938	0.005
***Overall***	***0.899***	***0.035***	***0.831–0.967***	***< 0.001***

**Fig. 2:**
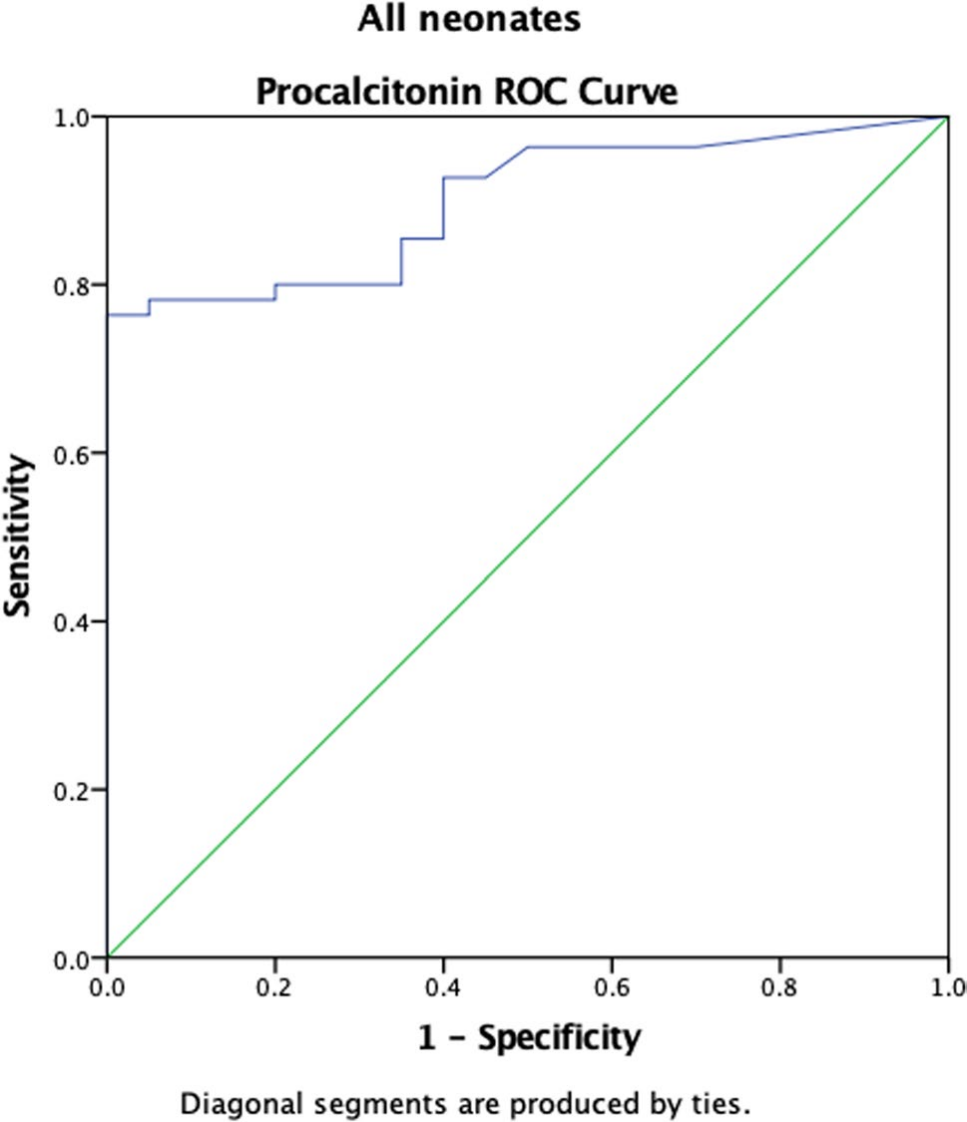
Procalcitonin ROC curve for all Neonates

In all neonates, the ROC curve demonstrated an area under the curve (AUC) of 0.899 (95% CI: 0.831–0.967; *p*< 0.001), indicating good overall discriminative ability of PCT in identifying culture-confirmed sepsis. This is shown in Fig. 2 below.

**Fig. 3:**
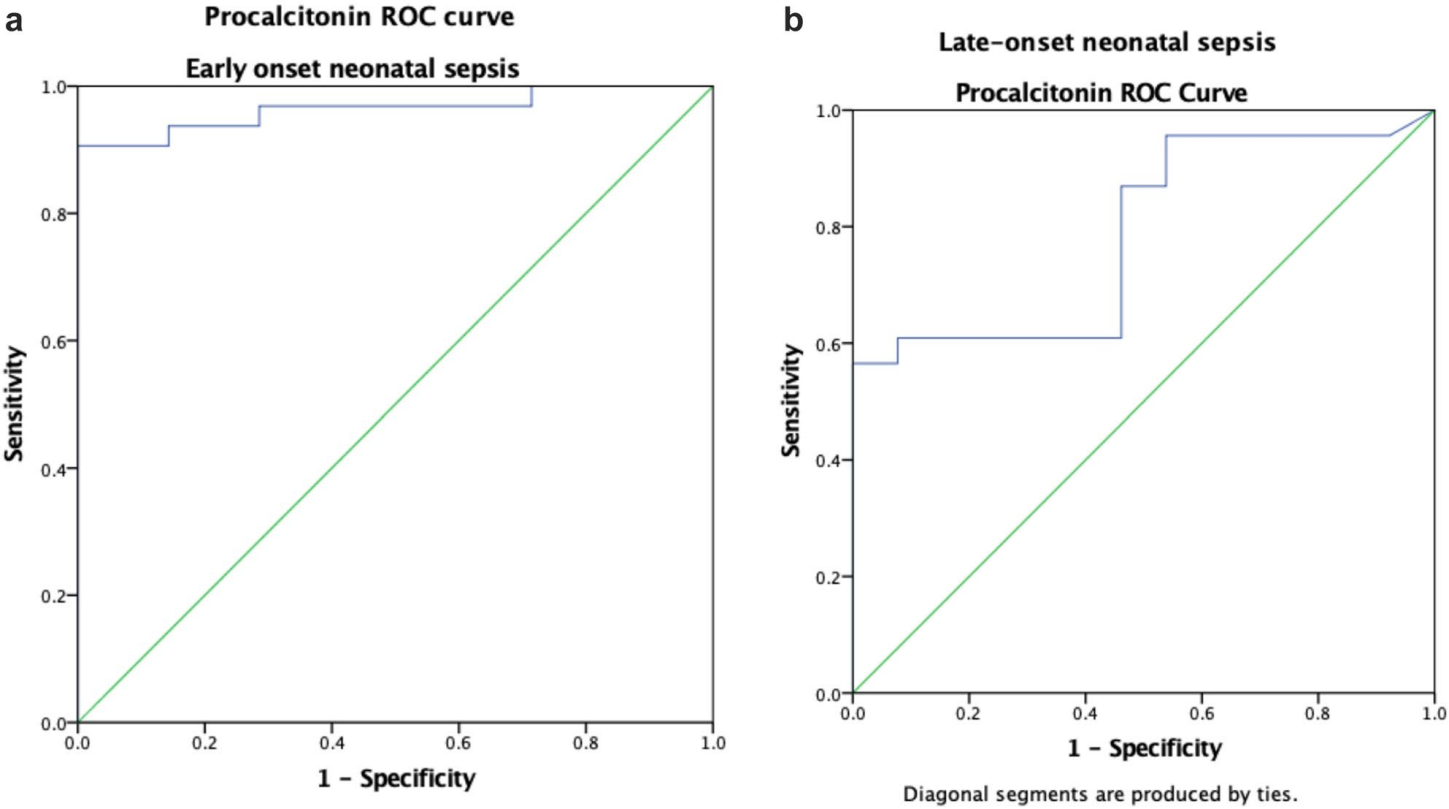
**a** Procalcitonin ROC curve for Early-onset neonatal sepsis. **b** Procalcitonin ROC curve for Late-onset neonatal sepsis

Subgroup analysis showed variation in diagnostic performance of PCT according to the timing of onset of sepsis. In neonates with early-onset neonatal sepsis (EONNS), the ROC curve (Fig. 3a) yielded an AUC of 0.964 (95%CI: 0.910–1.000; *p*< 0.001). The ROC curve (Fig. 3b) among those with late-onset neonatal sepsis (LONNS), showed a lower AUC of 0.788 (95% CI: 0.638–0.938; *p* = 0.005) emphasizing the need for complementary diagnostic approaches in older neonates.

### Blood culture isolates in study participants

Table 5 shows the distribution of organisms isolated from blood cultures in neonates with suspected sepsis. A total of 55 neonates had positive blood cultures, yielding 59 isolates, reflecting the presence of polymicrobial infections in some cases.

**Table 5:** Blood culture isolates in study participants with confirmed sepsis (*n*=55)

Variables	*n* = 55 Frequency	(%)
Blood culture (positive)		
Gram positive	20	36.4
Gram negative	35	63.6
Organisms cultured	55	
Escherichia coli	19	34.5
Staphylococcus aureus	13	23.6
Klebsiella pneumoniae	6	10.9
Pseudomonas spp	4	7.3
Coagulase-negative Staphylococcus	3	5.5
Micrococcus spp	3	5.5
Proteus spp	3	5.5
Enterobacter aerogenes	2	3.6
Candida spp	3	2.1
Enterococcus spp	1	1.8
Salmonella spp	1	1.3
Bacteroides spp	1	0.7

Gram-negative organisms predominated, accounting for 63.6% of isolates, while Gram-positive organisms represented 36.4%. Among Gram-negative bacteria, *Escherichia coli* were the most frequently isolated pathogen (34.5%), followed by *Klebsiella pneumoniae* (10.9%) and *Pseudomonas spp* (7.3%). Less common Gram-negative isolates included *Proteus spp* (5.5%), *Enterobacteraerogenes* (3.6%), *Salmonella spp* (1.3%), and *Bacteroides spp* (0.7%).

Gram-positive organisms were led by *Staphylococcus aureus* (23.6%**)**, which was the second most common overall isolate. Other Gram-positive bacteria included *Coagulase-negative Staphylococcus* (5.5%), *Micrococcus spp* (5.5%), and *Enterococcus spp* (1.8%).

Fungal isolates were rare, with *Candida spp* accounting for 2.1% of total isolates.

## Discussion

Reducing morbidity and mortality from neonatal sepsis requires prompt and accurate diagnosis with early initiation of appropriate antibiotic therapy; however, diagnostic uncertainty frequently leads to antibiotic overtreatment in neonates who presents with non-specific features of suspected sepsis [16]. Lately, measurement of procalcitonin has been reported as sensitive parameter for the early diagnosis of neonatal sepsis and evaluating its outcome [17]. The present study demonstrates that serum procalcitonin is a valuable diagnostic biomarker for neonatal sepsis in a Nigerian tertiary hospital; with its strong point being the ideal specificity of 100% observed at a cut-off value of ≥ 2.0 ng/mL. The key finding of this study is the excellent specificity and PPV (both 100%) of PCT at a cut-off of ≥ 2 ng/mL. This indicates that a PCT level above this threshold is a strong indicator of culture-proven sepsis, which is crucial for justifying antibiotic initiation in settings where culture results are delayed or unavailable. The lower sensitivity for LONNS (56.5%) at the cut-off value of ≥ 2 ng/mL signifies that a lower PCT level does not consistently rule out sepsis in this group, and clinical judgment must hold sway.

In the present study, the procalcitonin cut-off values of 0.5, 2, and 10 ng/mL were deliberately selected a priori, informed by previously published Nigerian studies such as Arowosegbe et al. [13] and Ezeh et al. [18] who have applied similar thresholds, demonstrating their clinical relevance within comparable resource-poor settings. The use of these predefined cut-offs therefore facilitates meaningful comparison with existing local and global evidence and reflects clinically pragmatic decision points, particularly for screening and rule-in diagnosis in settings where rapid microbiological confirmation is limited. Although, alternative data-driven approaches such as Youden’s Index [19] may identify statistically optimal thresholds by maximizing combined sensitivity and specificity, this method was not applied in the current study due to sample size considerations. Nonetheless, its application in larger, multicentre studies may further refine context-specific PCT cut-offs for neonatal sepsis.

Another significant finding in this study is that the blood culture positivity rate was 73.3% with majority (58.2%) being early-onset neonatal sepsis. This is similar to findings of 70.3% positivity rate reported by Opare-Asamoah et al. [20] in Ghana but higher than rates of 31.7%, 22% and 17.8% documented in Calabar [18], Abeokuta [13] and Ile-Ife [21] respectively. The higher rate recorded in the present study may be attributable to BacT/Alert blood culture system and ELISA method of sample analysis employed in the study.

When analysis was done at different cut-offs of PCT, the present study showed that all neonates with serum PCT 2 ng/mL had culture-positive sepsis corroborating findings in some other studies [22, 23] where serum PCT level of ≥ 2 ng/mL was the diagnostic threshold for identifying sepsis. At a cutoff of ≥ 0.5 ng/mL, sensitivity, specificity, PPV and NPV of serum PCT was 81.8%, 65%, 86.5% and 56.5% respectively. This is similar to a study by Arowosegbe et al. [13] in Abeokuta who documented comparable values of sensitivity and PPV rates of 89% and 84% respectively at a cutoff of ≥ 0.5 ng/mL, but with a lower specificity (23%) and higher NPV (83%). The lower NPV recorded by the current study may be due to inclusion of neonates with prior antibiotic exposure into the study, exposure that is documented to suppress bacterial load leading to increased false-negatives. Habib et al. [24] in Pakistan demonstrated higher rates of sensitivity, specificity and NPV which could be attributed to larger sample size. In contrast, Ezeh et al. [18] reported sensitivity, specificity, PPV and NPV rates of 68.4%, 29.3%, 31.0% and 66.7% which are lower than that we reported. This may be attributed to the conventional method of blood culture analysis used in their study [18]. As the cutoff value of serum PCT in our study increases, sensitivity decreases with increased specificity and PPV.

This study recorded sensitivity, specificity, PPV and NPV rates of 72.7%, 100%, 100% and 57.1% respectively at a cutoff of ≥ 2 ng/mL. These values indicate that this cut-off is more discriminatory for neonatal sepsis due to lower false-positive rates. Das et al. [22], Nellis et al. [23] and Arowosegbe et al. [13]. also documented the decrease in sensitivity with increase in specificity and PPV as the cut-off level of serum PCT increases.

The overall diagnostic accuracy of serum procalcitonin across all neonates at a cut-off level of ≥ 2 ng/mL was good with an AUC of 0.899 and is statistically significant. Our finding is comparable to reports by Rees et al. [25] in a systematic review and meta-analysis conducted in low- and middle-income countries, with an AUC of 0.87 (95% CI 0.70 to 0.92) but differs with reports of Ezeh et al. [18] who documented an AUC of 0.52 at a cut-off of 2 ng/mL. The higher AUC recorded in our study may be due to number of neonates recruited, heterogeneity of the study population and the Bact/Alert blood culture analysis used compared to other workers. Also, studies by Arowosegbe et al. [13] and Ezeh et al. [18] reported an area under the curve of 0.686 and 0.520 respectively at a lower cut-off of 0.5 ng/mL.

An important finding of our study is the apparent difference in the diagnostic performance of procalcitonin between EONNS and LONNS. The superior diagnostic performance of serum procalcitonin in early-onset neonatal sepsis observed in this study is supported by several findings from the results. Procalcitonin demonstrated a significantly higher area under the curve in EONNS compared to LONNS (0.964 vs. 0.788), indicating near-perfect diagnostic accuracy in early-onset disease. In addition, sensitivity was consistently higher in EONNS than LONNS at both evaluated cut-off values. Culture positivity was also higher among neonates with EONNS (82.1%) compared to LONNS (63.9%), suggesting a higher bacterial burden in early-onset disease. Furthermore, the predominance of Gram-negative organisms, particularly *Escherichia coli*, which are known to elicit a strong procalcitonin response, provides microbiological support for the enhanced performance of procalcitonin in EONNS. In contrast, the lower sensitivity observed in LONNS despite preserved specificity suggests a more heterogeneous inflammatory response in late-onset infections.

In this study, the microbiological profile is dominated by Gram-negative organisms especially *Escherichia coli*, which is consistent with findings in developing countries and aligns with some regional studies [22, 26, 27] However, it contrasts with reports identifying *Staphylococcus aureus* [28–30] and *Klebsiella pneumonia* as the predominant pathogens [31] These differences in bacterial isolates can be due to variations in regional microbial flora, differences in hospital infection control practices or demographic factors unique to the study population. Also, differences in sample size, design, and study area can further explain the discrepancy. These variations underscore the importance of local antimicrobial resistance surveillance and the serious nature of neonatal infections in our setting.

## Study limitations

The sample size, though adequate, was modest particularly for the LONNS subgroup. In particular, the relatively small number of neonates with late-onset neonatal sepsis resulted in reduced statistical power for this subgroup, and sensitivity estimates should therefore be interpreted with caution. The single-center design may limit generalizability. The significant proportion of neonates with prior antibiotic use likely reduced the blood culture positivity, affecting the calculated NPV of PCT; however, it helped to determine the diagnostic performance of PCT in poor resource where antibiotic use before presentation is high.

Other sepsis markers such as C-reactive protein, absolute neutrophil count, total leukocyte count, and micro-ESR were not uniformly available for all participants due to resource constraints. This limited direct comparison between procalcitonin and other inflammatory markers. In addition, inter-assay and intra-assay precision metrics were not independently validated locally, as procalcitonin measurements were performed using a commercial ELISA kit according to manufacturer specifications.

## Conclusion

Serum procalcitonin demonstrates high specificity for culture-confirmed neonatal sepsis at a cut-off of ≥ 2.0 ng/mL in the neonatal population, with better diagnostic performance in early-onset disease. These findings apply to the studied population and cut-off thresholds. Procalcitonin may serve as a useful rule-in biomarker to support timely antibiotic initiation in resource-limited settings, thereby improving antimicrobial stewardship without compromising patient safety. We recommend its integration into routine neonatal sepsis evaluation protocols in Nigerian hospitals, though larger, multi-center studies are warranted to validate these findings and establishing standardized diagnostic cut-off algorithms tailored to local epidemiological contexts.

## Data Availability

The datasets generated and analysed during the current study are available from the corresponding author upon reasonable request.

## Abbreviations

AUC: Area under the curve
EONNS: Early-onset neonatal sepsis
ELISA: Enzyme-linked immunosorbent assay
IQR: Interquartile range
IL-6: Interleukin 6
LONNS: Late-onset neonatal sepsis
NPV: Negative predictive value
PCT: Procalcitonin
PPV: Positive predictive value
ROC: Receiver Operating Characteristic curves
TNF-α: Tumor necrosis factor- alpha
